# Accurate Image Multi-Class Classification Neural Network Model with Quantum Entanglement Approach

**DOI:** 10.3390/s23052753

**Published:** 2023-03-02

**Authors:** Farina Riaz, Shahab Abdulla, Hajime Suzuki, Srinjoy Ganguly, Ravinesh C. Deo, Susan Hopkins

**Affiliations:** 1 Commonweatlh Scientific and Industrial Research Organisation, Sydney, NSW 2000, Australia; 2 UniSQ Collage, University of Southern Queensland, Brisbane, QLD 4000, Australia; 3 School of Mathematics, Physics and Computing, University of Southern Queensland, Springfield, QLD 4300, Australia

**Keywords:** artificial intelligence, artificial neural network, intelligent transportation system, quantum computer, quantum computing, quantum machine learning, traffic signs

## Abstract

Quantum machine learning (QML) has attracted significant research attention over the last decade. Multiple models have been developed to demonstrate the practical applications of the quantum properties. In this study, we first demonstrate that the previously proposed quanvolutional neural network (QuanvNN) using a randomly generated quantum circuit improves the image classification accuracy of a fully connected neural network against the Modified National Institute of Standards and Technology (MNIST) dataset and the Canadian Institute for Advanced Research 10 class (CIFAR-10) dataset from 92.0% to 93.0% and from 30.5% to 34.9%, respectively. We then propose a new model referred to as a Neural Network with Quantum Entanglement (NNQE) using a strongly entangled quantum circuit combined with Hadamard gates. The new model further improves the image classification accuracy of MNIST and CIFAR-10 to 93.8% and 36.0%, respectively. Unlike other QML methods, the proposed method does not require optimization of the parameters inside the quantum circuits; hence, it requires only limited use of the quantum circuit. Given the small number of qubits and relatively shallow depth of the proposed quantum circuit, the proposed method is well suited for implementation in noisy intermediate-scale quantum computers. While promising results were obtained by the proposed method when applied to the MNIST and CIFAR-10 datasets, a test against a more complicated German Traffic Sign Recognition Benchmark (GTSRB) dataset degraded the image classification accuracy from 82.2% to 73.4%. The exact causes of the performance improvement and degradation are currently an open question, prompting further research on the understanding and design of suitable quantum circuits for image classification neural networks for colored and complex data.

## 1. Introduction

The theory of machine learning is an important subdiscipline in both artificial intelligence and statistics, with roots in artificial neural networks and artificial intelligence research since the 1950s [1]. Data processing using quantum devices is known as quantum computing. Because operations can be performed on numerous states simultaneously, the capacity of quantum states to be in a superposition can significantly speed up computation in terms of complexity in a broader machine learning context. Several quantum machine learning (QML) variations of classical models have recently been developed, including quantum reservoir computing (QRC) [2], quantum circuit learning (QCL) [3,4,5], continuous variable quantum neural networks (CVQNNs) [6], quantum kitchen sinks (QKSs) [7,8,9], quantum variational classifiers [10,11], and quantum kernel estimators [12,13]. Recent literature surveys on QML are available [14,15,16]. We note that the main approach taken by the community consists in formalizing problems of interest as variational optimization problems and using hybrid systems of quantum and classical hardware to find approximate solutions [15]. The intuition is that by implementing some subroutines on classical hardware, the requirement of quantum resources is significantly reduced, particularly the number of qubits, circuit depth, and coherence time, making the quantum algorithms suitable to be implemented on noisy, intermediate-scale quantum (NISQ) devices [15]. Recent examples in this direction include the work by Arthur and Date, who proposed a hybrid quantum-classical neural network architecture where each neuron is a variational quantum circuit [17], and the work by Sagingalieva et al., who proposed a combination of classical convolutional layers, graph convolutional layers, and quantum neural network layers to improve on drug–response prediction over a purely classical counterpart [18].

Among the many proposals to combine classical machine learning methods with quantum computing, the method proposed by Henderson et al. in [19] has the advantage of being implementable in quantum circuits with a smaller number of qubits with shallow gate depths and can be applied to more practical applications. This method utilizes quantum circuits as transformation layers to extract features for image classification using convolutional neural networks (CNNs). These transformation layers are called quanvolutional layers, and the method is herein referred to as the quanvolutional neural network (QuanvNN).

An important question raised was whether the features produced by the quanvolutional layers could increase the accuracy of the machine learning models for classification purposes. Henderson et al. attempted to address this question by applying randomly created quantum circuits and comparing the classification accuracy of the QuanvNN with the results obtained by a conventional CNN. The results did not show a clear advantage in terms of classification accuracy over the classical model [19]. The QuanvNN was further updated in [20], implemented on quantum computer hardware (Rigetti’s Aspen-7-25Q-B quantum processing unit), and evaluated in a satellite imagery classification task. However, the image classification accuracy of the QuanvNN did not improve compared with that of the conventional CNN.

The implementation of the QuanvNN on a software quantum computing simulator, PennyLane [21], was provided by Mari [22]. Mari’s implementation of QuanvNN differs from that of Henderson et al. in at least two aspects. Firstly, Mari’s implementation combined a quanvolutional layer with a neural network (NN) instead of CNN. Secondly, the output of the quantum circuit (a set of expectation values) was directly fed into the following neural network layer, while the output of the quantum circuit was converted into a single scalar value using a classical method in the original QuanvNN proposal by Henderson et al. In Mari’s implementation, 50 training and 30 test images from the Modified National Institute of Standards and Technology (MNIST) dataset (a handwritten 10 class 10-digit dataset [23]) were applied and tested. No clear improvement in the classification accuracy of QuanvNN over NN was observed in [22].

In this paper, we first show that a QuanvNN using a randomly generated quantum circuit (four qubits with 20 single-axis rotations and 10 controlled NOTs (CNOTs), extending Mari’s implementation from using one random layer to five random layers) improves the image classification accuracy of a classical fully connected NN against MNIST and the Canadian Institute for Advanced Research 10 class (CIFAR-10) dataset (photographic 10 class image dataset [24]) from 92.0% to 93.0% and from 30.5% to 34.9%, respectively. We then propose a new model, termed Neural Network with Quantum Entanglement (NNQE), using a strongly entangled quantum circuit (four qubits with 20 three-axis rotations and 20 CNOTs) combined with Hadamard gates, instead of random quantum circuits. Our newly proposed NNQE further improves the image classification accuracy against MNIST and CIFAR-10 to 93.8% and 36.0%, respectively. These improvements were obtained using a quantum circuit consisting of only four qubits without introducing any additional parameters to the optimizing machine learning process. Unlike other QML methods, the proposed method does not require optimization of the parameters inside the quantum circuits; hence, it requires only limited use of the quantum circuit. Given the small number of qubits and relatively shallow depth of the proposed quantum circuit, the proposed method is well suited for implementation in noisy intermediate-scale quantum computers.

However, using QuanvNN or the proposed NNQE degrades the image classification performance when applied to a more complicated German Traffic Sign Recognition Benchmark (GTSRB) dataset (43 class real-life traffic sign images [25]) in comparison with the classical NN accuracy from 82.2% to 71.9% (QuanvNN) and to 73.4% (NNQE). Nevertheless, we note that NNQE produced improved image classification accuracy over QuanvNN from 71.9% to 73.4%. The exact causes of the performance improvement and degradation are currently an open question, prompting further research on the understanding and design of suitable quantum circuits for image classification neural networks for colored and complex data. We note that a similar result of QuanvNN not improving the image classification accuracy of NN when tested against GTSRB was also recently reported in [26], which is consistent with our findings.

The remainder of this paper is organized as follows: Section 2 presents the methodology for the proposed model. The details of our experiment are provided in Section 3. The results and discussion are presented in Section 4, followed by conclusions in Section 5.

## 2. Methods

For the implementation of QuanvNN, readers are referred to [22], noting that the number of random layers was increased from 1 to 5. This results in the use of a quantum circuit with 20 random single-axis rotations and 10 CNOTs with QuanvNN.

**Figure 1:**
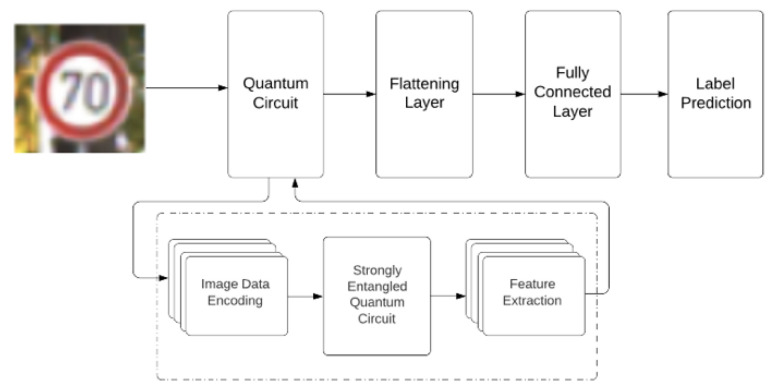
A flowchart of the proposed NNQE model.

Figure 1 shows a flowchart of our proposed NNQE model. We assume that the input image is a two-dimensional matrix of size *m*-by-*m* and the pixel value *x* follows 0 ≤ *x* ≤ 1. The extension to a multichannel pixel image is expected to be straightforward. A section of size *n*-by-*n* is extracted from the input image, where *n* = 2. An extension of *n* > 2 will be left for further study.

Given *n* = 2, we use a 4-qubit quantum circuit. The four qubits are initialized in the ground state, and the four-pixel values are then encoded using RY with *θ* = π*x* as in (1).

(1) $$\mathrm{RY}=\left[\begin{array}{cc} \cos\theta /2 & -\sin\theta /2\\ \sin\theta /2 & \cos\theta /2 \end{array}\right]$$

The outputs from RY gates are fed to the quantum circuit.

NNQE uses four Hadamard gates, 20 three-axis rotations, and 20 CNOTs. One Hadamard gate is applied to each qubit immediately after encoding. The gates are grouped into five layers, with each layer consisting of four three-axis rotations and four CNOTs. Three-axis rotation is applied to each qubit within the layer. The rotation angles were chosen randomly and uniformly between 0 rad and 2 π rad. The four CNOTs within each layer connect the qubits randomly, but without overlap. The Hadamard and CNOT gates can be described mathematically as in (2) and (3), respectively.

(2) H=12  [111−1]

(3) $$\mathrm{CNOT}=\left[\begin{array}{c} \begin{array}{cc} 1 & 0\\ 0 & 1 \end{array}\;\begin{array}{cc} \;\;\;0 & 0\\ \;\;\;0 & 0 \end{array}\\ \begin{array}{cc} 0 & 0\\ 0 & 0 \end{array}\;\begin{array}{cc} \;\;\;0 & 1\\ \;\;\;1 & 0 \end{array} \end{array}\right]$$

The outputs from the measurement operations are given as expectation values between −1 and 1 and form the output features.

**Figure 2:**
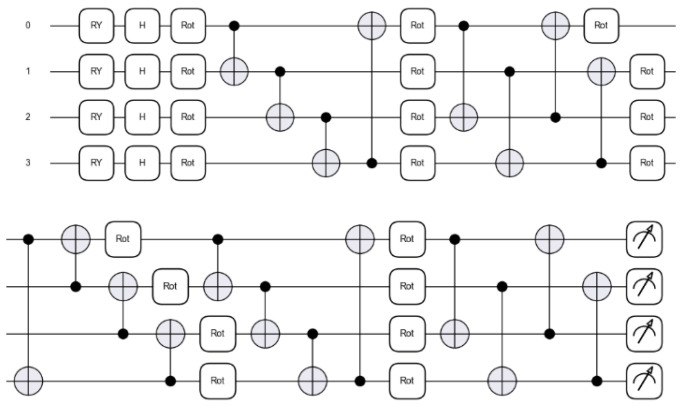
The quantum circuit architecture of the proposed NNQE Circuit model with 5 layers.

The output features are transformed into a one-dimensional vector using a flattening layer, as shown in Figure 1. The output of the flattening layer is connected to the fully connected layer to classify and predict the image labels for testing model learning. The dotted box in Figure 1 is expanded in detail in Figure 2. The circuit is expanded into multiple rotations and CNOTs in the expectation it will achieve better feature extraction than that of the random circuit. In particular, the design of the quantum circuit was inspired by the Circuit 15 in [27] which was found to retain high expressibility with a strong entangling capability. In addition, an extra layer of the Hadamard gates was added by us by trial, which showed further performance improvements.

## 3. Experiment

The method proposed in this study was implemented on a quantum computing simulator using Python (version 3.7.0) and PennyLane libraries (release 0.27.0) [21]. The random quantum circuit and strongly entangled quantum circuit were implemented using PennyLane’s built-in RandomLayers and StronglyEntanglingLayers functions. Unless otherwise stated, the Adam optimizer and a batch size of 128 were used to train the network. Table 1 summarizes the parameters of the three image datasets used in this experiment. The method was implemented on a MacBook Pro (Intel Core i7 2.5 GHz CPU). Processing data for MNIST for NNQE, for example, took approximately two days each.

### 3.1. Testing Dataset MNIST

**Figure 3:**
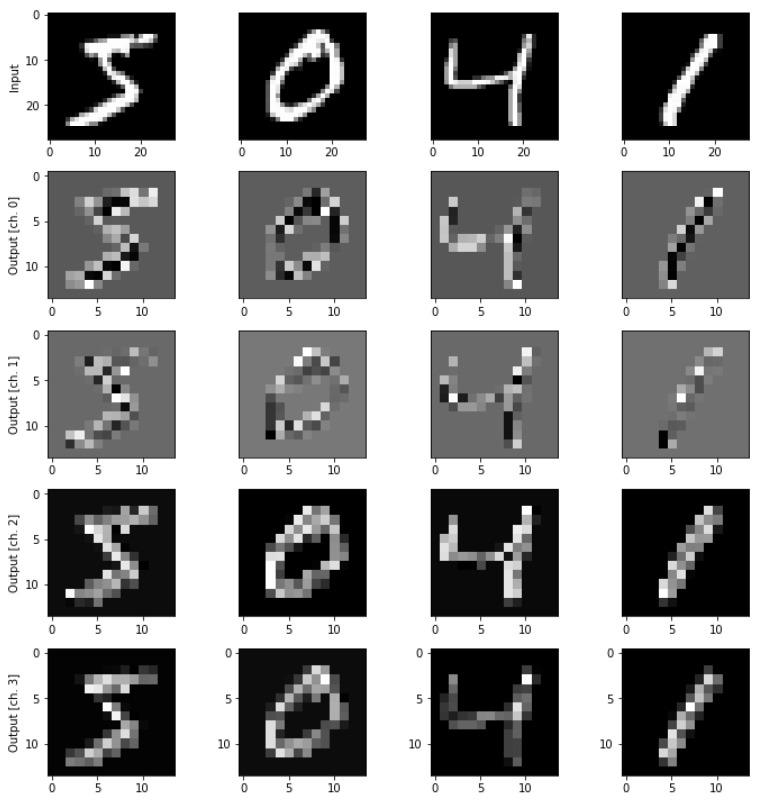
Example MNIST dataset images and corresponding output features using NNQE circuit model.

The MNIST dataset [23] consists of 60,000 training and 10,000 testing images of handwritten digits from 0 to 9. Each image measures 28 × 28 pixels. The original images are grayscale with values between 0 and 255, which were scaled by dividing them by 255. Figure 3 shows an example of the MNIST dataset images and the corresponding output features obtained using NNQE circuit.

### 3.2. Testing Dataset CIFAR-10

**Figure 4:**
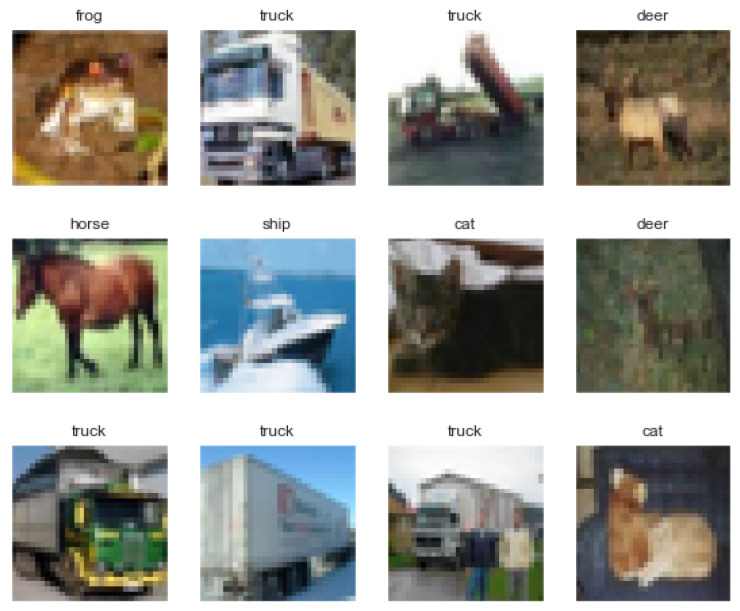
Example visualization of CIFAR-10 colored dataset images.

**Figure 5:**
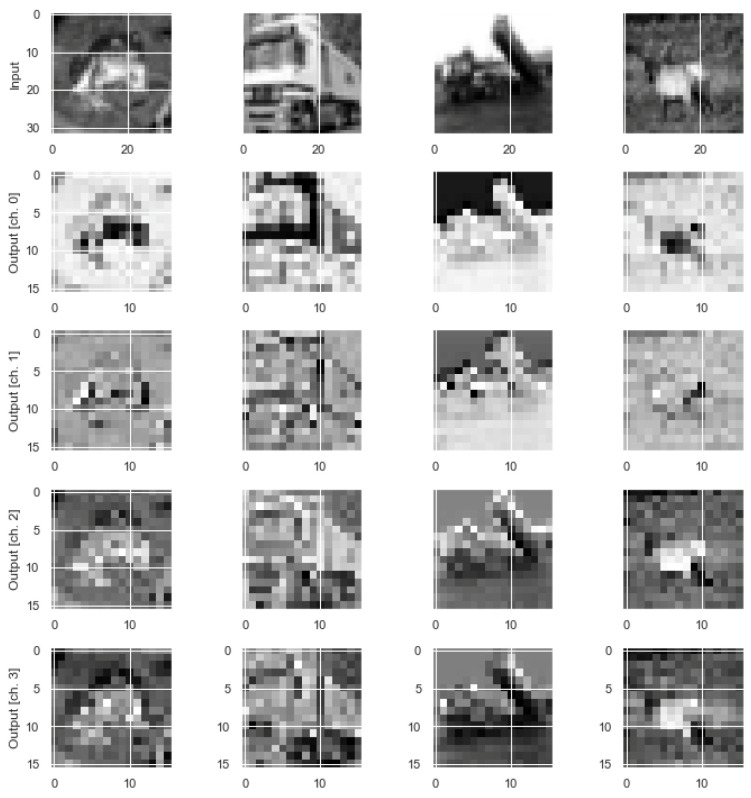
Example CIFAR-10 dataset images and corresponding output features using NNQE circuit.

The CIFAR-10 dataset [24] consists of 50,000 training images and 10,000 testing images. Photographic images are colored and consist of ten classes. The original images are in RGB color, which were converted into grayscale between 0 and 255 and then scaled by dividing them by 255. Examples of CIFAR-10 dataset images are shown in Figure 4. Figure 5 shows an example of the original CIFAR-10 dataset images and the corresponding output features obtained using an NNQE circuit.

### 3.3. Testing Dataset GTSRB

**Figure 6:**
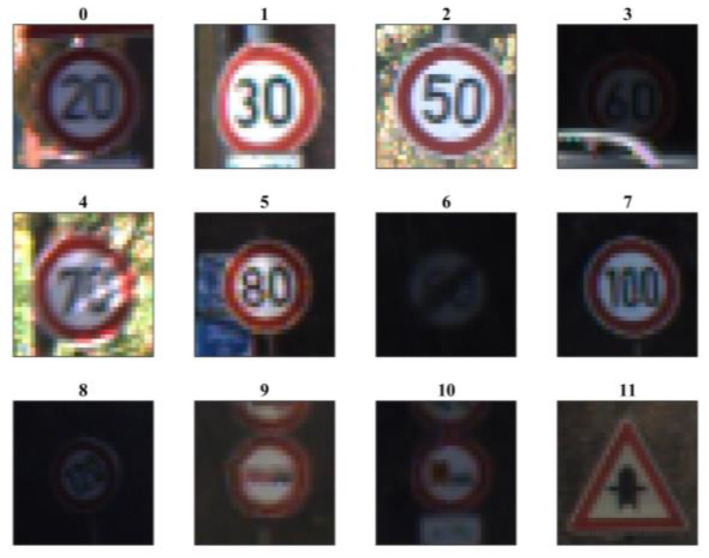
Example GTSRB dataset images. (Digits indicate class labels.)

**Figure 7:**
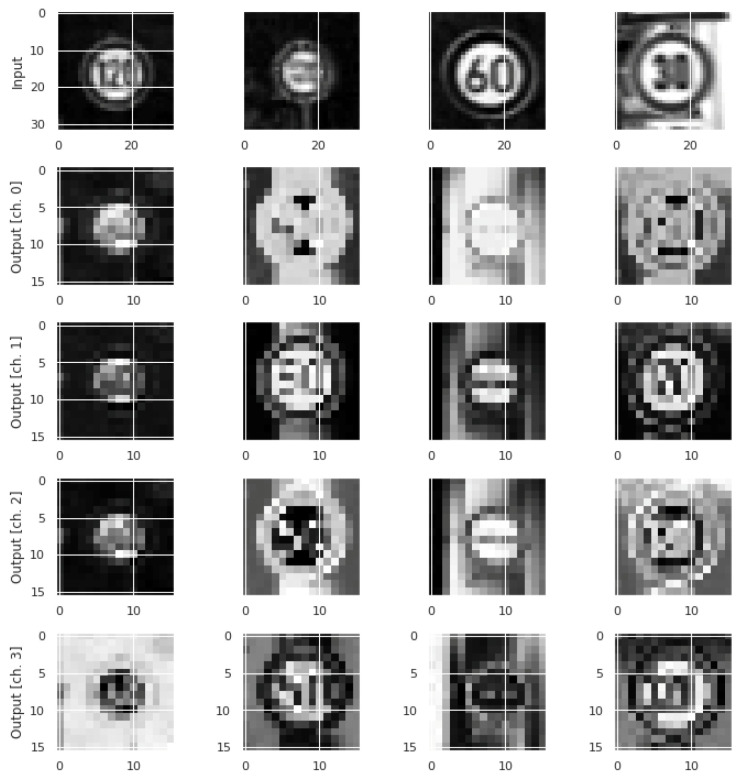
Example GTSRB dataset images and corresponding output features using NNQE circuit.

The GTSRB dataset [25] consists of 34,799 training and 12,630 test images of 43 classes of traffic signs captured from actual use under various conditions. These images were captured at night, during rainy weather, and in fog-based atmospheric environments under various illumination conditions, which could make it challenging for any machine to learn concealed features from dark and relatively unclear images. The original dataset has image sizes varying between 15 × 15 pixels and 222 × 193 pixels. As suggested by Sermanet and LeCun in [28], the images were scaled to 32 × 32 pixels. The original images are in RGB color, which were converted into grayscale between 0 and 255 and then scaled by dividing them by 255. Examples of the GTSRB dataset images are shown in Figure 6, whereas the original and corresponding output features using the NNQE circuit are shown in Figure 7.

## 4. Results and Discussion

**Figure 8:**
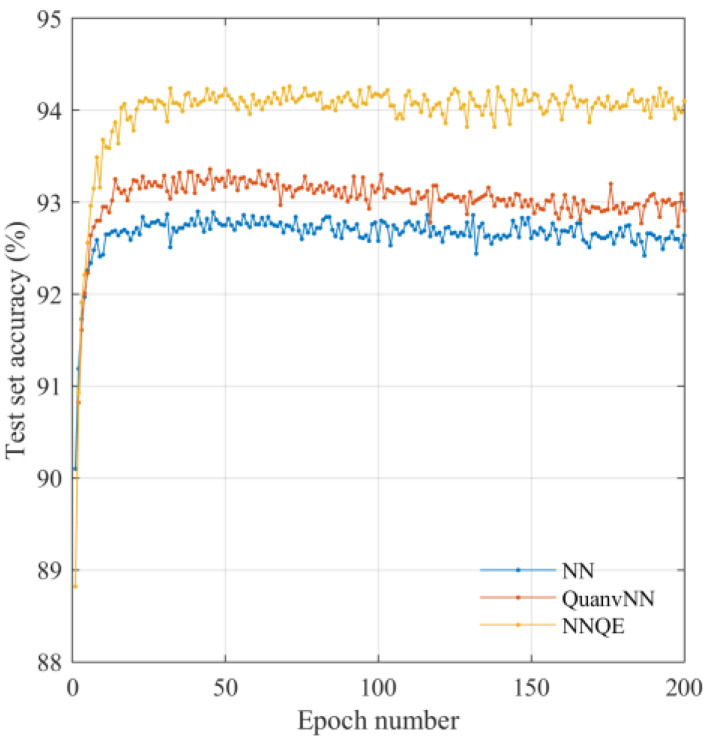
Test set accuracy against MNIST dataset images.

Figure 8 shows the variation in the classification accuracy of the test set as a function of the training epoch using the MNIST dataset. As shown in Figure 8, QuanvNN improves the accuracy of the test set over the classical NN. The performance was further improved by the NNQE circuit. Again, we emphasize that this improvement was obtained without introducing any additional optimizing parameters in the machine learning process.

**Figure 9:**
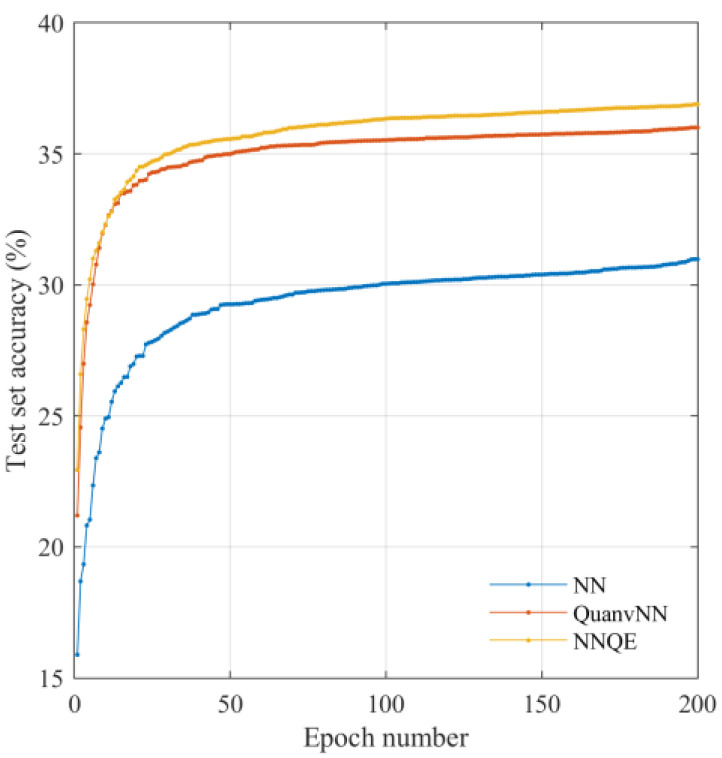
Test set accuracy against CIFAR-10 dataset images.

Figure 9 shows the variation in the accuracy of the test set as a function of the training epoch using the CIFAR-10 dataset. A large improvement was obtained by the application of the QuanvNN over the classical NN, with a further improvement obtained by the application of the NNQE circuit.

**Figure 10:**
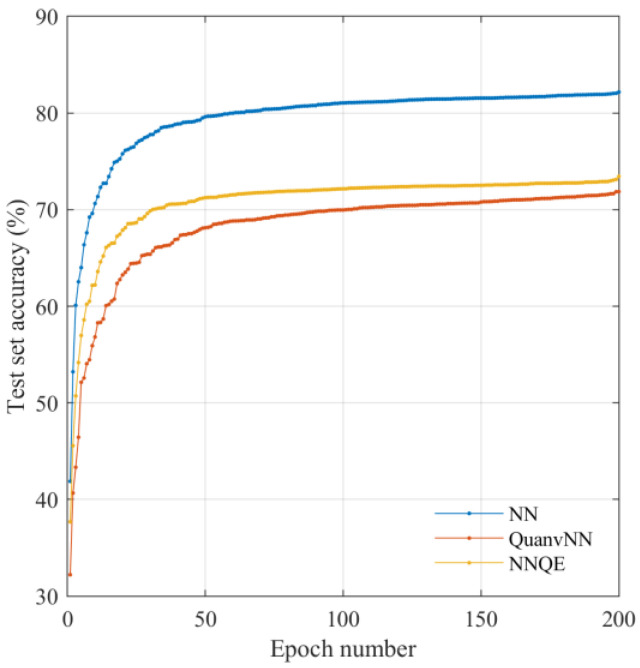
Test set accuracy against GTSRB dataset images.

Figure 10 shows the variation in the accuracy of the test set using the GTSRB dataset. Unlike the cases using the MNIST and CIFAR-10 datasets, the test set accuracy obtained using the QuanvNN was reduced compared with that of the classical NN. However, the proposed NNQE circuit outperforms the QuanvNN, as shown in Figure 10.

We note that in each case of the MNIST, CIFAR-10, and GTSRB datasets, other classical methods, such as CNNs, which are algorithmically more complex but can be implemented efficiently on modern processors, can in practice produce a higher image classification accuracy than that by our proposed NNQE method. However, the benefit of our proposed NNQE method is to observe that the application of the quantum circuit can improve the image classification accuracy over a classical method. Understanding the exact causes of this observation is expected to lead a better design of the quantum circuit that is more beneficial in practice in the future. However, the exact cause of this phenomenon is currently unknown and requires further investigation. We believe one plausible reason could be the better correlations between the image pixels that may be enhanced owing to the strong entanglement between the qubits, thereby leading to an overall improvement in accuracy. A summary of the results is presented in Table 2.

**Figure 11:**
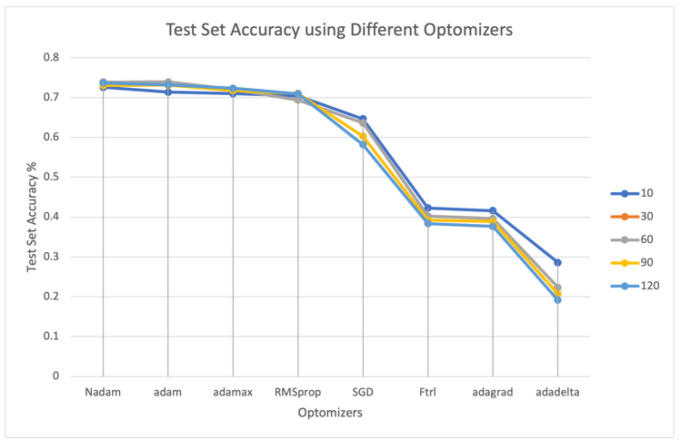
Test set accuracy using different optimizers and batch sizes against GTSRB. The batch size is listed in the legend.

To investigate the characteristics of the proposed NNQE, eight optimizers and five batch sizes were tested using the GTSRB dataset. These optimizers were used to run the model and have different effects on the model execution and training. The following are the models used to check the performance efficiency of our proposed NNQE circuit: Adam, AdaDelta, RMSProp, Adagrad, AdaMax, SGD, Nadam and FTRL. Figure 11 shows test set accuracy using the different optimizers and batch sizes against GTSRB.

It is evident from Figure 11 that the Nadam-based optimizer algorithm performs better than all other optimizers used in this study. For the different batch sizes tested in Figure 11, the results show only a small difference among the best-performing optimizers using a wide range of batch sizes (10, 30, 60, 90, and 120).

## 5. Conclusions and Future Directions

In this study, we developed a new NNQE method and investigated the image classification performance using three different well-known image datasets. As shown in Table 2, the testing accuracy against MNIST (handwritten digits) was improved from 92.0% by the classical NN to 93.0% by the previously proposed QuanvNN, and further to 93.8% by our proposed NNQE. Similarly, the testing accuracy against CIFAR-10 (colored images) was improved from 30.5% by the classical NN to 34.9% by QuanvNN, and further to 36.0% by NNQE. Both MNIST and CIFAR-10 had 10 distinct classes. While the exact cause of this is not yet clear and requires further investigation, one plausible reason could be the better correlations between the image pixels that may be enhanced owing to the strong entanglement between the qubits, thereby leading to an overall improvement in accuracy. However, the performance of the proposed model was degraded when applied to real-life, complex, colored images of traffic signs (GTSRBs), which have 43 classes in comparison with the classical NN. This is shown in Table 2 as follows: The testing accuracy against GTSRBs by the classical NN was found to be 82.2%, which was reduced to 71.9% by the previously proposed QuanvNN. The testing accuracy against GTSRBs was improved from 71.9% to 73.4% by our proposed NNQE. However, this is still a reduction in the testing accuracy against GTSRB from 82.2% achieved by the classical NN. This indicates that further development of the NNQE model may be necessary for relatively larger classes in more complex datasets, such as real-life traffic signs and GTSRBs. We also tested different optimizers for the proposed model to demonstrate the efficacy of NNQE model further. The results showed that Nadam-based optimizers produced the most optimal results. This is perhaps attributable to the Nadam algorithm being an extension of Adam optimizers, which add Nesterov’s Accelerated Gradient (NAG), or Nesterov momentum, to provide an improved type of momentum for the search procedure. Future research could also include increasing the number of qubit sizes from four, as well as investigating the indicators of performance improvements, or their relative degradation, in comparison with classical NN. These studies could involve proposing new methodologies for designing quantum circuits to build on the present study and tests with more complex datasets with larger classes or concealed data features.

## Figures and Tables

**Table 1:** Parameters of image datasets used in experiment.

	MNIST	CIFAR-10	GTSRB
**Image Size**	28 × 28	32 × 32	32 × 32
**Number of color channel**	1	3	3
**Number of classes**	10	10	43
**Number of training set images**	60,000	50,000	34,799
**Number of testing set images**	10,000	10,000	12,630

**Table 2:** Converged training and testing accuracy.

	Classical NN	QuanvNN	NNQE
Training Accuracy against MNIST	93.5%	94.2%	95.1%
Testing Accuracy against MNIST	92.0%	93.0%	93.8%
Training Accuracy against CIFAR-10	34.1%	41.2%	42.0%
Testing Accuracy against CIFAR-10	30.5%	34.9%	36.0%
Training Accuracy against GTSRB	96.2%	94.8%	97.5%
Testing Accuracy against GTSRB	82.2%	71.9%	73.4%

## Data Availability

The data presented in this study are available on request from the corresponding author.
